# The effect of active exoskeleton support with different lumbar-to-hip support ratios on spinal musculoskeletal loading and lumbar kinematics during lifting

**DOI:** 10.1017/wtc.2024.7

**Published:** 2024-12-23

**Authors:** Niels P. Brouwer, Ali Tabasi, Feng Hu, Idsart Kingma, Wietse van Dijk, Mohamed Irfan Mohamed Refai, Herman van der Kooij, Jaap H. van Dieën

**Affiliations:** 1 Department of Human Movement Sciences, Vrije Universiteit Amsterdam, Amsterdam Movement Sciences, Amsterdam, The Netherlands; 2 TNO, Leiden, The Netherlands; 3 Department of Biomechanical Engineering, University of Twente, Enschede, The Netherlands

**Keywords:** Occupational exoskeleton, trunk-support exoskeleton, manual material handling, musculoskeletal disorders, lumbo-pelvic rhythm

## Abstract

While active back-support exoskeletons can reduce mechanical loading of the spine, current designs include only one pair of actuated hip joints combined with a rigid structure between the pelvis and trunk attachments, restricting lumbar flexion and consequently intended lifting behavior. This study presents a novel active exoskeleton including actuated lumbar and hip joints as well as subject-specific exoskeleton control based on a real-time active low-back moment estimation. We evaluated the effect of exoskeleton support with different lumbar-to-hip (L/H) support ratios on spine loading, lumbar kinematics, and back muscle electromyography (EMG). Eight healthy males lifted 15 kg loads using three techniques without exoskeleton (NOEXO) and with exoskeleton: minimal impedance mode (MINIMP), L/H support ratio in line with a typical L/H net moment ratio (R0.8), lower (R0.5) and higher (R2.0) L/H support ratio than R0.8, and a mechanically fixed lumbar joint (LF; simulating hip joint-only exoskeleton designs).

EMG-driven musculoskeletal model results indicated that R0.8 and R0.5 yielded significant reductions in spinal loading (4–11%, p < .004) across techniques when compared to MINIMP, through reducing active moments (14–30%) while not affecting lumbar flexion and passive moments. R2.0 and LF significantly reduced spinal loading (8–17%, p < .001; 22–26%, p < .001, respectively), however significantly restricted lumbar flexion (3–18%, 24–27%, respectively) and the associated passive moments.

An L/H support ratio in line with a typical L/H net moment ratio reduces spinal loading, while allowing normal lifting behavior. High L/H support ratios (e.g., in hip joint-only exoskeleton designs) yield reductions in spinal loading, however, restrict lifting behavior, typically perceived as hindrance.

## 1. Introduction

Low-back pain (LBP) remains the leading cause of disability worldwide (Hartvigsen et al., 2018; Hoy et al., 2014; Wu et al., 2020). LBP incidence and its socioeconomic consequences are expected to increase in the coming decades (Hartvigsen et al., 2018), and an important LBP risk factor is mechanical spine loading (Coenen et al., 2013, 2014; da Costa & Vieira, 2010; Griffith et al., 2012; Hoogendoorn et al., 2000; Norman et al., 1998; Punnett et al., 1991).

Back-support exoskeletons have been suggested as a tool to reduce LBP risks (de Looze et al., 2016; Kermavnar et al., 2021) and are classified as passive (support based on storing and releasing energy produced by the user’s motion), active (support based on actuators) (Kermavnar et al., 2021), or, as recently suggested, semi-active (combined passive and active actuation; Crea et al., 2021). Active exoskeletons can provide support linked to the user’s intent and interaction with the environment through their control, and are therefore potentially more versatile (Toxiri et al., 2019).

Typically, studies evaluating active exoskeletons report a reduction in back muscle electromyography (EMG) amplitude (de Looze et al., 2016; Kermavnar et al., 2021), suggesting reduced muscle – and possibly spinal – loading. However, an EMG amplitude decrease may also be related to an increase in lumbar flexion (Kingma et al., 2022; Madinei and Nussbaum, 2023), leading to a load shift from active to passive back tissues, implying that EMG reduction does not necessarily coincide with a reduction in spinal loading. Consequently, the evaluation of the effect of exoskeletons on low-back loading may require biomechanical models estimating, for example, active/passive joint moments and spinal compression forces (Kingma et al., 2022; Koopman, Kingma, et al., 2019; Koopman, Näf, et al., 2020; Koopman, Toxiri, et al., 2019; Madinei & Nussbaum, 2023; Moya-Esteban et al., 2022).

While two previous studies on an actuated exoskeleton reported a decrease in peak compression forces (Koopman, Toxiri, et al., 2019; Lazzaroni et al., 2020), lumbar flexion range of motion was considerably reduced (on average 33%; Koopman, Toxiri, et al., 2019), typically experienced as hindering normal lifting behavior (Näf et al., 2018). This lumbar kinematics restriction may arise from the exoskeleton design involving a single bilateral actuated joint approximately aligned with the hip joints in the sagittal plane, combined with a rigid structure between the pelvis and trunk attachments (Koopman, Toxiri, et al., 2019; Toxiri et al., 2018). This single degree-of-freedom (DOF) characteristic is also commonly observed in other active exoskeletons (Chen et al., 2018; Heo et al., 2020; Hussain et al., 2020; Hyun et al., 2020; Ko et al., 2018; Miura et al., 2018; Toxiri et al., 2018; Walter et al., 2023; Xiang et al., 2023; Yamanaka et al., 2021; Zhang & Huang, 2018), and may fail to accommodate the variation in kinematic lumbar-to-hip (L/H) flexion ratio across users and tasks (Granata and Sanford, 2000; Laird et al., 2018; Takahashi & Yamaji, 2020). Two previous studies presented active exoskeletons including one additional bilateral low-lumbar (Schwartz et al., 2023) or multiple additional lumbar and thoracic (Lanotte et al., 2020) actuated DOFs. While Schwartz et al. (2023) indicated an additional DOF may affect lifting kinematics, they did not report details about the torque generated at each actuated DOF. Lanotte et al. (2020) indicated that in theory the hip support was twice the support at the lumbar and thoracic levels, however, did not determine the magnitude of support and its effect on spinal loading. Thus, in active back-support exoskeletons, the effect of an additional actuated DOF and consequently the between-DOF support ratio on spinal loading remains to be investigated.

We developed a novel active exoskeleton including (1) two pairs of bilateral actuated joints, to allow separate support for lumbar and hip flexion/extension, (2) motors with a sufficient torque generation capacity, and (3) exoskeleton control providing support as a percentage of the moment actively generated by the back muscles to counteract the moment caused by the gravitational force acting on the upper body. Note that support based on only the active moment avoids counterproductive support when moments are generated by passive tissues (Tabasi et al., 2020).

The present study aimed to evaluate the effect of exoskeleton support with different L/H support ratios on spine loading, lumbar kinematics, and back muscle EMG during squat, stoop, and free lifting. As a first step, we evaluated the effect of wearing the exoskeleton without support by comparing a without-exoskeleton condition (NOEXO) to a condition with the exoskeleton set to minimal impedance mode (MINIMP). In the second step, we evaluated five different exoskeleton conditions: (1) MINIMP, considered as the reference condition; (2) an L/H support ratio equal to an estimate of a typical L/H ratio during lifting (Toussaint et al., 1992); a (3) lower and (4) higher L/H support ratio when compared to (2); and (5) a mechanically fixed lumbar and actuated hip joint, simulating previous active exoskeleton designs including one bilateral actuated hip joint and a rigid trunk structure. For the latter condition, we expected to find a reduction in lumbar flexion relative to the other conditions, irrespective of lifting technique. In squat and free lifting, we expected for all exoskeleton support conditions a reduction in peak spinal loading and back muscle EMG relative to MINIMP. In stoop lifting, we expected more variance in reduction of peak spinal load given the between-participant variation in lumbar and hip flexibility, with some participants probably approaching or reaching flexion relaxation (Toussaint et al., 1995), resulting in active moments approaching or reaching zero.

## 2. Methods

### 2.1. Exoskeleton

#### 2.1.1. Structure

**Figure 1.**
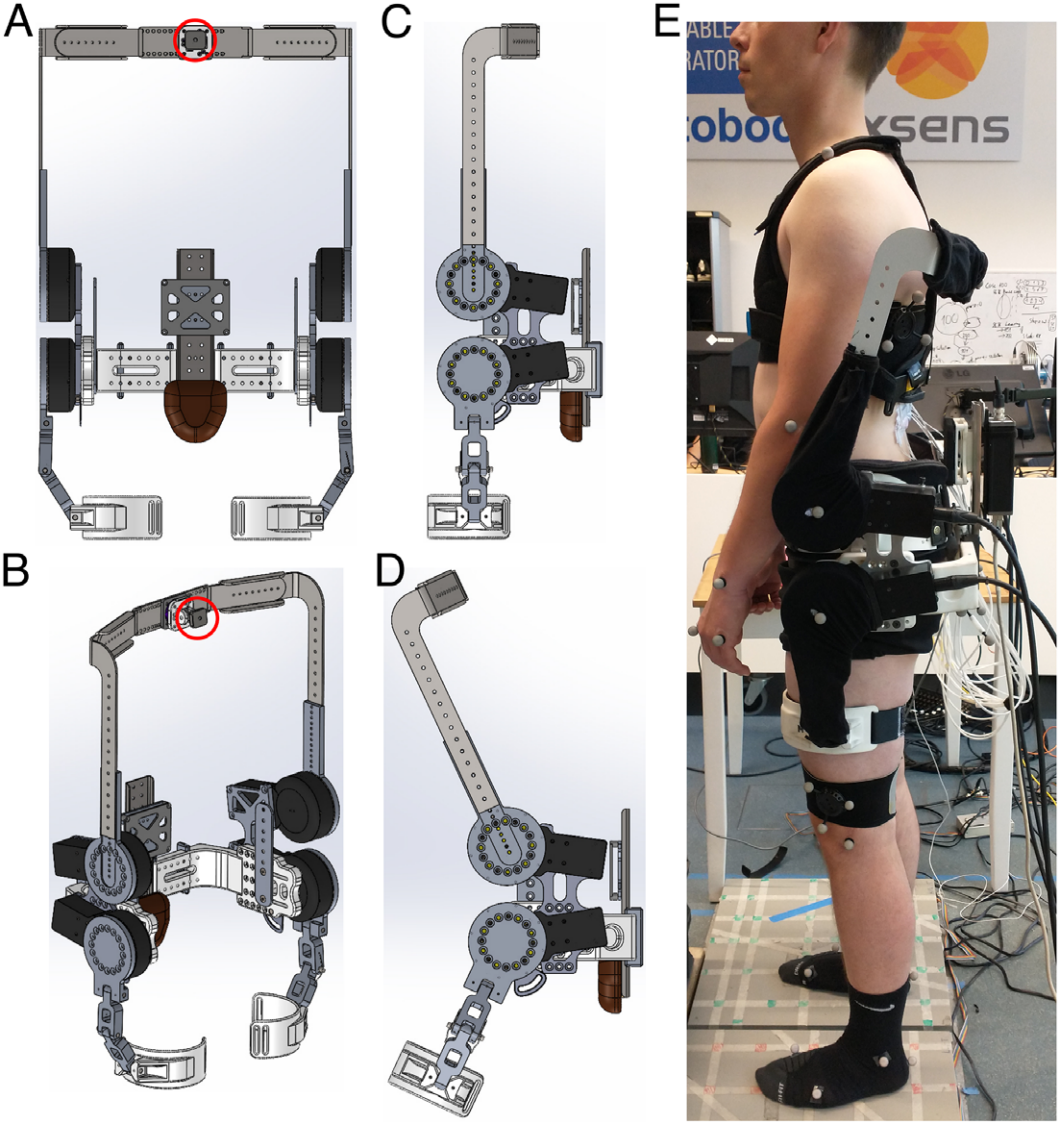
Front (A), 3D (B) and side (C, D) view of exoskeleton structure. The thorax vest (attachment to structure depicted with red circle), pelvis brace and padding, and thigh straps and padding are not displayed here. (E) Side view of participant wearing the exoskeleton.

The exoskeleton (designed and developed by the University of Twente and Delft University of Technology; weight: approx. 16.5 kg) contained four motors (Bacchus V3, TU Delft: weight: 1.5 kg; max torque: 100 Nm; max velocity: 60 rpm), two bilaterally at the level of the right/left hip joint (hip motors) and two bilaterally approximately at the level of L3 (lumbar motors). The motors were connected using wires to an external pc and power module. Their torque and joint angle were internally measured using torque sensors and encoders at 1000 Hz. The exoskeleton contained an adjustable thorax vest (Laevo FLEX, Laevo, the Netherlands), an adjustable pelvis brace, and two bilateral thigh wraps (Figure 1).

#### 2.1.2. Control model

When considering a top-down approach, the net moment around L5S1 (Mnet) involves the net moment due to the gravitational force and (angular) acceleration acting on the upper body (Mnet_ub_) and the net moment due to external forces (e.g., the load when lifting) acting on the upper body (Mnet_ext_) (see equation 1). Mnet is counteracted by actively (Mact; i.e., trunk extensor muscles) and passively (i.e., through elasticity of back tissues) generated moments (Mpas) (see equation 2). Mpas is determined by the lumbar flexion angle (van Dieën and Kingma, 2005). The actively generated moment can be further split into the moment related to the gravitational force and (angular) acceleration acting on the upper body (Mact_ub_) and external forces (Mact_ext_) (see equation 2).
(1)

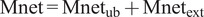



(2)

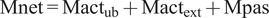

In this study, the support provided by the hip and lumbar motors relied on a real-time estimation of Mact_ub_. Mact_ub_ was computed by subtracting the estimated Mpas from the estimated Mnet_ub_ (see equation 3). As can be derived from equations 1 to 3, the control model did not account for Mact_ext_.
(3)

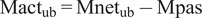

Mnet_ub_ was estimated using the trunk sagittal plane inclination angle measured using an inertial measurement unit (IMU) attached to the trunk (at 27.5% from MPSIS to C7, approx. at the height of the T12 the spinous process; Faber et al., 2013) and the participants’ body mass and trunk length. Mpas was estimated using an a priori estimation of the subject-specific Mpas-lumbar flexion relationship using a lumbar flexion range-of-motion trial. This non-linear relationship (see van Dieën and Kingma, 2005) was determined using Mnet_ub_ and lumbar flexion at full flexion. In this procedure, we assumed a lumbar flexion angle of 20° as optimum angle for the trunk extensor muscles, defining the onset of Mpas, and an Mnet_ub_ at full flexion only generated by Mpas. The lumbar flexion angle was based on the relative orientation of the trunk IMU to a pelvis IMU (attached over the sacrum). Depending on the condition, a certain percentage of Mact_ub_ was set as desired support (i.e., command torque) for the hip and lumbar motors, separately. Note that, per motor, the eventual generated exoskeleton torque was the sum of the command torque and the torque associated with MINIMP.

### 2.2. Population and procedure

#### 2.2.1. Population

Eight healthy male participants with no history of low-back pain volunteered for this study (mean ± std age: 27 ± 3 years; height: 180 ± 5 cm; weight: 73.4 ± 6.4 kg) and signed informed consent prior to the experiment, approved by the local ethical committee of the University of Twente (reference number: 230181). We recruited only male participants in view of the considerable mass of the exoskeleton.

#### 2.2.2. Preparation

**Figure 2.**
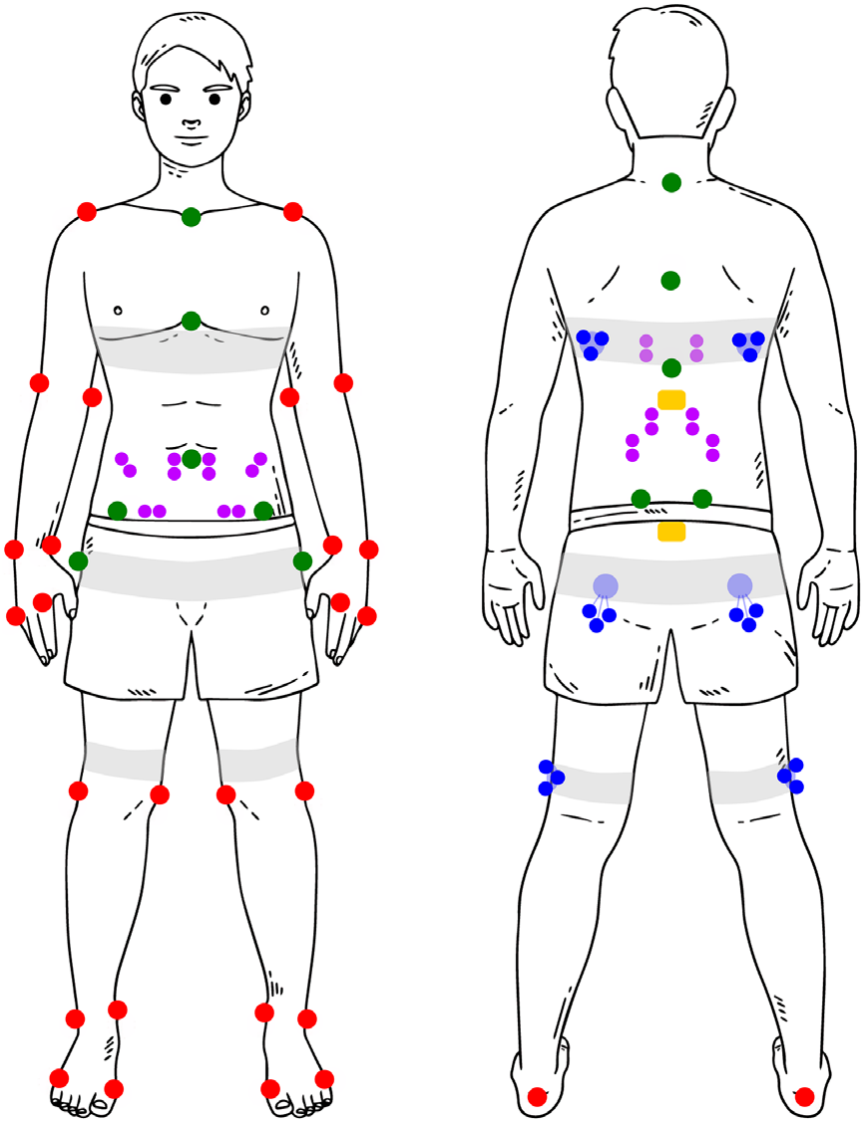
Schematic overview of IMU (**yellow**), EMG (**purple**), and reflective marker placement (red, blue, green). In **red**: single markers recorded during all trials; **blue**: cluster-markers recorded during all trials; **green**: single markers removed for exoskeleton trials (due to occlusion/interference with exoskeleton; their position was calculated during post-processing based on cluster-markers); **grey**: elastic bands.

First, the participants’ anthropometric data were obtained (circumference of body segments, body height and mass, and trunk length). Based on these data, the exoskeleton was fitted to the participant. After careful skin preparation, bipolar EMG electrodes were attached bilaterally over the longissimus thoracis pars lumborum, longissimus thoracis pars thoracis, iliocostalis lumborum, rectus abdominis, external oblique, and internal oblique muscles (Kingma et al., 2010). Participants performed symmetric and asymmetric maximum isometric contractions of the trunk extensor and abdominal muscles (multiple per muscle group) to obtain the maximum voluntary contraction (MVC) for each muscle (McGill, 1991). Next, while wearing the exoskeleton in MINIMP mode, the anatomical neutral posture, a 6 m walking trial, and a lumbar flexion range-of-motion trial (passively hanging down while standing with extended knees) were recorded to define the neutral orientation for both IMUs, align the IMUs with their respective segment (Rispens et al., 2014), and calibrate the exoskeleton control model, respectively. Then, a total of 40 single reflective markers were attached to the pelvis, abdomen, thorax, and left/right feet, shanks, thighs, upper arms, forearms, and hands to obtain 3D full-body kinematics (Figure 2). Four markers were attached laterally to the centres of the exoskeleton motors to obtain 3D exoskeleton joint centre position. Using elastic bands, six reflective cluster-markers were attached to the thorax (two, bilaterally below the scapulae), pelvis (two, bilaterally below the sacrum), and left and right thigh. After recording the anatomical neutral reference posture, several single markers were removed since, while wearing the exoskeleton, these markers would be occluded (C7, T6, T10, xiphoid, sternum, Navel left/right ASIS, left/right PSIS, left/right greater trochanter; Figure 2). During post-processing their trajectories were calculated using the cluster-markers and a projection procedure (Cappozzo et al., 1995).

#### 2.2.3. Lifting and exoskeleton conditions

Participants performed 5 NOEXO and 27 lifting trials with exoskeleton. From these 27 trials, the first 6 were familiarization trials: squat, stoop, or free technique and MINIMP or R0.8 (see Table 1 for explanation of the abbreviations and definition of the conditions). For the current study, 15 of the other 21 exoskeleton trials, as well as 3 of the 5 NOEXO trials were analyzed (Table 1). The two excluded NOEXO trials (0 kg and 7.5 kg box weight using free technique) were only used for fitting the EMG-driven subject-specific model (section 2.4.1).

**Table 1.** Conditions considered in this study. Each exoskeleton condition was recorded using squat, stoop, and free technique with a box weight of 15 kg. NOEXO: without-exoskeleton condition; MINIMP: with exoskeleton set to minimal impedance mode; R0.8: lumbar/hip (L/H) ratio equal to an estimate of a typical L/H ratio during lifting (Toussaint et al., 1992); R0.5 and R2.0 representing a lower and higher L/H support ratio compared to R0.8; LF: mechanically fixed lumbar motor with only hip support (simulating previous active exoskeleton designs).

Box weight (kg)	Exoskeleton condition	Technique	Lumbar support (%Mact_ub_)	Hip support (%Mact_ub_)	Lumbar/Hip support ratio
15	NOEXO	Free/Squat/Stoop	–	–	–
15	MINIMP	Free/Squat/Stoop	0	0	–
15	R0.8	Free/Squat/Stoop	40	50	0.8
15	R0.5	Free/Squat/Stoop	25	50	0.5
15	R2.0	Free/Squat/Stoop	50	25	2
15	LF	Free/Squat/Stoop	Fixed	50	–

In each trial, participants lifted the box (weight 15 kg, box handles height: approx. 20 cm) from force plate height (i.e., floor height), with one trial including three lifting cycles (one cycle: lowering without box, lifting the box, lowering with box, and returning to upright standing without box) with a trial duration between 25 and 30 seconds to control the pace. Six exoskeleton conditions were considered and separately recorded using lifting techniques squat, stoop, and free (see Table 1). Trials were grouped in four blocks: (1) NOEXO; (2) MINIMP, R0.8, and R0.5; (3) R2.0; and (4) LF. Within each block the trial order was counterbalanced (i.e., for blocks 1, 3, 4: random technique order; for block 2: random technique and exoskeleton condition order). Blocks 3 and 4 were always recorded after block 2 since pilot testing indicated that the exoskeleton could shift relative to the body in these conditions. For the first and last 4 participants, the NOEXO trials (i.e., block 1) were recorded last and first, respectively. In 3 participants, the box was elevated by 15 cm during LF (participant 1: squat, free and stoop; participant 2: free and stoop; participant 7: stoop), since participants were not able to reach the box. In participant 5, R2.0 and LF with stoop technique could not be recorded due to too much shifting of the exoskeleton relative to the body.

### 2.3. Instrumentation

Surface EMG was recorded at 2048 Hz (Porti, TMSi, the Netherlands) and raw IMU (Xsens DOT, Movella, the Netherlands) orientation data was streamed with 60 Hz in real-time via Bluetooth to the exoskeleton PC. Full-body and box kinematics were collected at 100 Hz by a 12-camera motion capture system (Qualisys Medical AB, Sweden). Ground reaction forces and moments were recorded at 1600 Hz using two force plates (AMTI, USA).

### 2.4. Data analysis

Kinematic and kinetic data were filtered using a 5 Hz zero-lag second-order Butterworth low-pass filter. During data analysis the marker-based sagittal plane kinematics of the pelvis appeared to be affected by soft-tissue artefacts related to the Gluteus muscles and were therefore replaced by the IMU-based pelvis inclination. The EMG data were band-pass filtered using a 30-400 Hz zero-lag second-order Butterworth filter (Redfern et al., 1993), full-wave rectified, normalized to MVC, and one-way low-pass filtered using a 2.5 Hz fourth-order Butterworth to obtain the linear envelope (Potvin, 1996). Using a dynamic 3D linked segment model and bottom-up inverse dynamics, net L5S1 reaction forces and moments around L5S1 were computed (Kingma et al., 1996). For with-exoskeleton trials, exoskeleton mass was added to the pelvis centre of mass, since the bulk of the mass was carried by the pelvis.

#### 2.4.1. Low-back compression force and moments

A subject-specific EMG-driven musculoskeletal model was used to compute low-back compression force and active, passive, and total muscle moments at the level of the L5S1 joint (van Dieën and Kingma, 2005). The model includes musculoskeletal anatomy and EMG-muscle force relationships, comprising active force-length, force-velocity, and passive force-length relationships, and is driven by abdominal and low-back EMG linear envelope signals and lumbar sagittal plane kinematics. The model was calibrated to the individual using anthropometric data and seven parameters describing the subject-specific muscle contractile properties: (1) gain factor for the EMG–force relationship, (2, 3) scaling factor for the width and offset factor of the active force–length relationship, (4, 5) scaling factor and offset factor for the passive force–length relationship, and (6, 7) two scaling factors for the active force–velocity relationship of eccentric and concentric contractions, respectively (van Dieën and Kingma, 2005). These seven parameters were determined in an optimization minimizing the error between the inverse dynamics-based net moment (Mnet) and the total muscle moment generated by the model (Mmusc). For calibration, all NOEXO trials were used: 15 kg box weight using free, squat, and stoop technique, and 0 kg and 7.5 kg box weight using free technique. To evaluate model fit, the RMSE between Mnet and Mmusc was computed across all calibration trials. To evaluate model performance, the *R* ^2^ and RMSE between Mnet minus exoskeleton lumbar torque (Msubject) and Mmusc were computed across all included trials except LF (Table 1; no exoskeleton lumbar torque was measured for LF).

#### 2.4.2. Instant of peak compression force

Since peak loading has been suggested to be a major contributor to cumulative low-back loading (Coenen et al., 2012) and a risk factor for LBP (Coenen et al., 2013), the instance of peak L5S1 compression force (Fcomp) was selected to evaluate the effect of wearing the exoskeleton without support and exoskeleton support with different L/H ratios on spinal loading. Employing the model, for all included trials (Table 1), peak Fcomp was identified for each first half of each lifting cycle (i.e., lowering without and lifting with box), resulting in three instances of peak Fcomp per trial (note that each trial included three full lifting cycles). For these instances of peak Fcomp, the active moment (Mact), passive moment (Mpas), Mmusc, lumbar flexion, average back muscle linear envelope (back muscle EMG), Mnet, and exoskeleton lumbar and hip torque were obtained. Per variable, these three values were averaged to obtain one mean value per trial.

#### 2.4.3. Continuous measures

For R0.8, R0.5, and R2.0, to evaluate whether the intended L/H support ratio was eventually generated, per trial, the mean generated L/H support ratio was computed by obtaining the ratio of the average lumbar over average hip support measured by the torque sensors embedded in the motors. For MINIMP, R0.8, R0.5, R2.0, and LF trials, exoskeleton-anatomical joint alignment error was evaluated using the RMSE of the norm of the sagittal plane vector between the respective joint centres: left/right exoskeleton lumbar joints and L5S1, left/right exoskeleton hip joints and left/right hip joints. The left and right RMSE were averaged to obtain one average error for lumbar and hip joint centre alignment. For all conditions, the average actual L/H Mnet ratio across each trial was computed with the ratio of the average L5S1 joint Mnet over the average hip joint Mnet.

### 2.5. Statistics

In the first step, to evaluate the effect of wearing the exoskeleton without providing support on peak Fcomp, Mact, Mpas, Mmusc, lumbar flexion, back muscle EMG, and Mnet, two-way repeated measures ANOVAs were performed, with technique (squat, stoop, free) and exoskeleton (NOEXO, MINIMP) as within-subject factors. We used the Greenhouse–Geisser epsilon to determine sphericity. If epsilon was ≥0.75, the Huynh–Feldt correction was selected. For epsilon <0.75, the Greenhouse–Geisser correction was selected (Girden, 1992). In case of a significant interaction effect, per technique, a paired *t*-test with Bonferroni correction was used to identify differences between NOEXO and MINIMP.

In the second step, to evaluate the effect of exoskeleton support with different L/H ratios on peak Fcomp, Mact, Mpas, Mmusc, lumbar flexion, back muscle EMG, Mnet, and lumbar and hip joint centre alignment error, two-way ANOVAs were performed, with subject as random factor, and technique (squat, stoop, free) and exoskeleton (MINIMP, R0.8, R0.5, R2.0, LF) as fixed factors. In case of a significant main effect of exoskeleton condition, with no significant interaction effect, paired *t*-tests with Bonferroni correction were used to identify differences between exoskeleton conditions. In case of a significant interaction effect, per technique, a one-way ANOVA was performed with subject as random factor and with exoskeleton (MINIMP, R0.8, R0.5, R2.0, LF) as fixed factor. In case of a significant main effect, paired *t*-tests with Bonferroni correction were used to identify differences between exoskeleton conditions.

## 3. Results

Across participants, the RMSE between Mnet and Mmusc for the calibration trials ranged from 9.0 to 19.4 Nm and, for all trials, the *R* ^2^ and RMSE between Msubject and Mmusc ranged from 0.75 to 0.93 and 18.8 to 26.3 Nm, respectively. Across all conditions, at peak Fcomp the range of L/H Mnet ratios for squat, stoop, and free techniques were 0.86–0.92, 0.81–0.83, and 0.85–0.88, respectively (Figure S1), indicating that our a priori choice for R0.8 as the reference support condition was appropriate.

While no significant effects of only wearing the exoskeleton (without support) were found on spinal loading (peak Fcomp, and Mmusc and back muscle EMG at peak Fcomp), a load shift from active to passive tissues due to increased lumbar flexion and a slight increase in Mnet was observed when wearing the exoskeleton (Table S1; Figure S2).

### 3.1. L/H support ratio: intended versus generated

**Figure 3.**
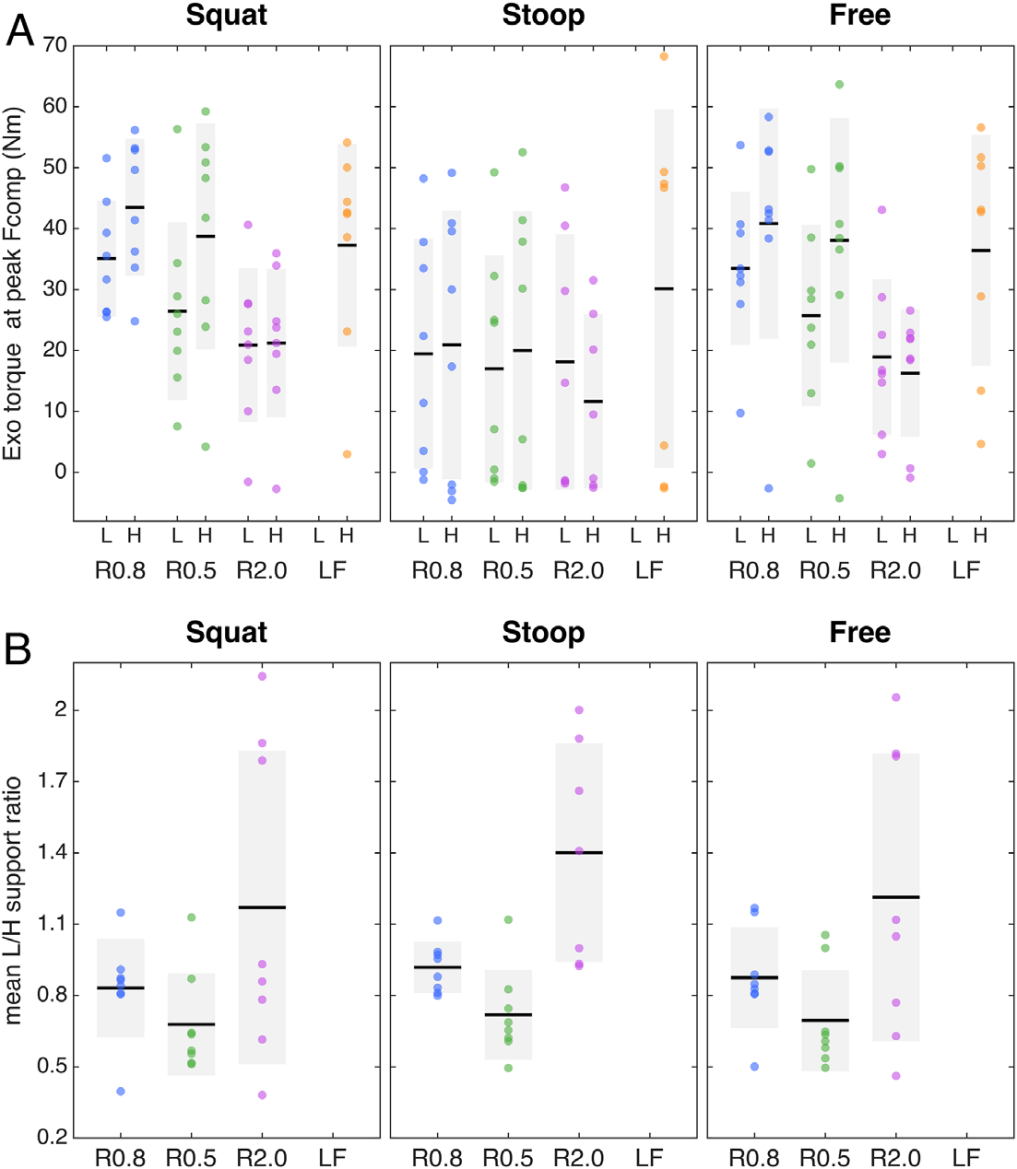
(A) Generated lumbar (L) and hip (H) support at peak compression force (Fcomp), and (B) mean generated L/H support ratio across trial, for the conditions involving exoskeleton support, per lifting techniques. Note that for LF no lumbar torque and no L/H support ratio was included since the lumbar exoskeleton joint was mechanically fixed. The black line and grey rectangle depict the mean and standard deviation, respectively.

**Figure 4.**
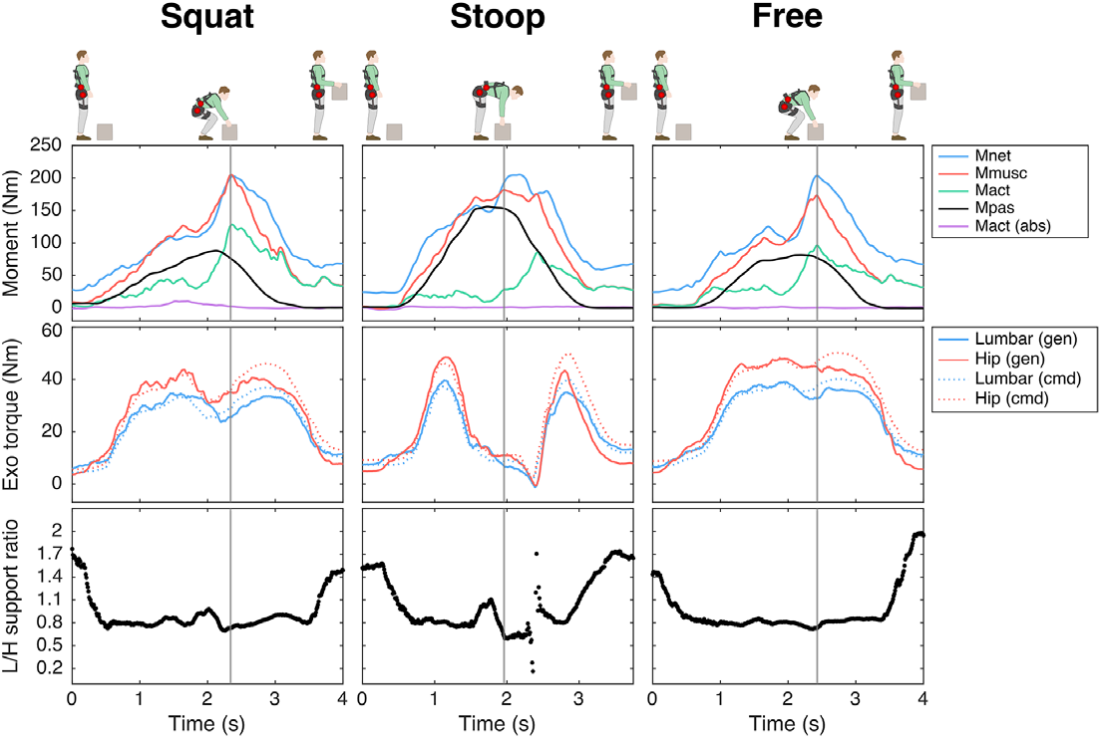
Example (single participant) of time series of the net L5S1 moment (Mnet), total muscle moment (Mmusc), active moment (Mact), passive moment (Mpas), lumbar flexion, active abdominal moment (Mact (abs)), lumbar and hip generated (gen) and command (cmd) exoskeleton (Exo) torque, and generated lumbar-to-hip (L/H) support ratio across lifting techniques (squat, stoop, free) for the exoskeleton support condition R0.8. The instant of peak compression force is depicted with the vertical grey line.

For squat and free lifting considerable exoskeleton support was provided in most participants at peak Fcomp, whereas for the stoop technique in about half of the participants the exoskeleton support approached or reached zero at peak Fcomp, indicating the control model predicted an Mnet_ub_ (almost) entirely generated by passive tissues (Figure 3A, see Figure 4 for a time series example, note the high Mpas during stoop lifting). The other participants received support during stoop lifting, as at least part of Mnet_ub_ was estimated to result in active force produced by the trunk extensor muscles. Note that for all techniques, and most prominently for stoop lifting, the maximum lumbar and hip support across trials was on average higher (Figure S3) than the average support at peak Fcomp (Figure 3A).

**Figure 5.**
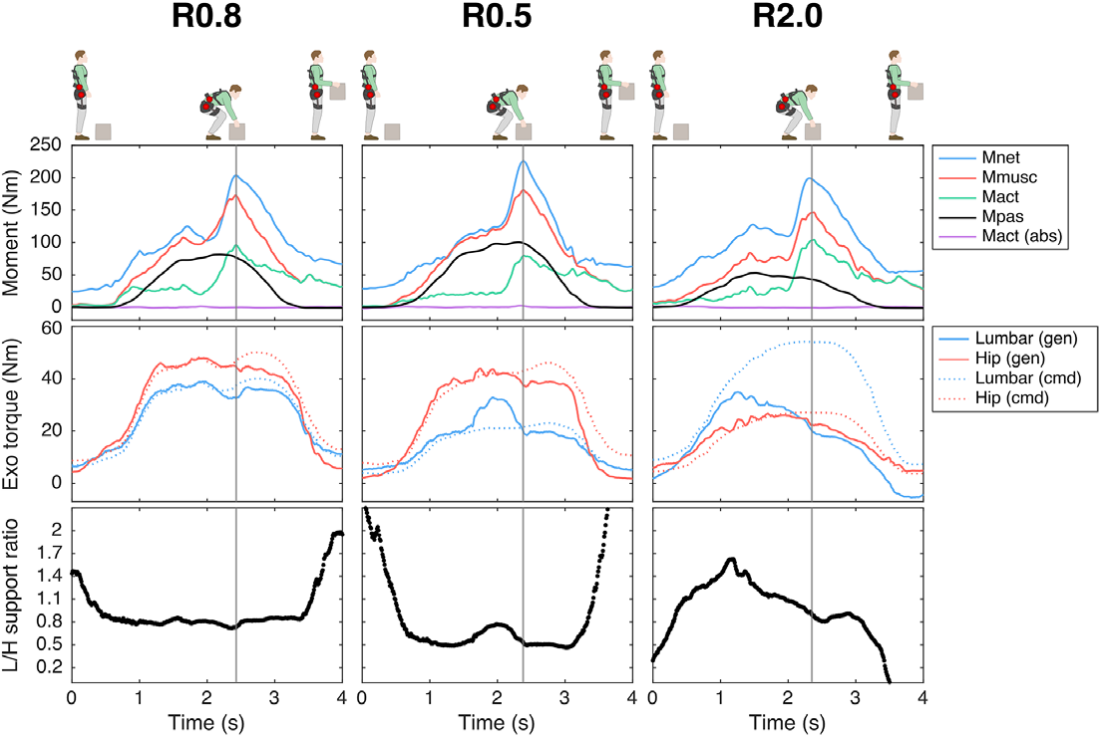
Example (single participant) of time series of the net L5S1 moment (Mnet), total muscle moment (Mmusc), active moment (Mact), passive moment (Mpas), lumbar flexion, active abdominal moment (Mact (abs)), lumbar and hip generated (gen) and command (cmd) exoskeleton (Exo) torque, and generated lumbar-to-hip (L/H) support ratio during free lifting for the exoskeleton support conditions: R0.8, R0.5, R2.0. Note that, while satisfactory in R0.8 and R0.5, in R2.0 the difference between generated and command torque (i.e., torque tracking) was greater due to reduced transfer of support as a result of exoskeleton pelvis brace shifting. The instant of peak compression force is depicted with the vertical grey line.

The mean generated L/H support across participants is presented per condition and technique in Figure 3B (see Figure 5 for a time series example). Across lifting techniques, for R0.8, the mean generated L/H support ratio was in line with the intended L/H support ratio (on average for squat, stoop, free lifting: 0.83, 0.92, 0.87, Figure 3B; intended 0.8) and with the mean L/H Mnet ratio (Figure S1). R0.5 yielded a slightly higher mean generated L/H support ratio than intended (on average for squat, stoop, free: 0.68, 0.72, 0.69, Figure 3B); intended: 0.5). In R2.0, the exoskeleton generally failed to generate the intended lumbar and (to a lesser extent) hip support at peak Fcomp (on average 20 Nm for both lumbar and hip; Figure 3A) and consequently the L/H support ratio was lower than intended (mean generated L/H support ratio for squat, stoop, free: 1.2, 1.4, 1.2, Figure 3B; intended: 2). This is supported by Figure 5, which indicates that, while the magnitude of generated support and L/H support ratio during the initial phase of the lift approached the intended values, in R2.0 a drop in generated lumbar torque can be observed before peak Mmusc (i.e., strongly correlated to peak Fcomp; van Dieën and Kingma, 2005), whereas the command torque did not prescribe a reduction in support. In addition, in line with the average generated lumbar and hip support (Figure 3A), the difference between generated and command torque was considerably higher for the lumbar than hip motors (Figure 5). In R2.0 and LF (no lumbar torque could be measured since the lumbar joint was fixed), we observed upwards shifting of the pelvis brace relative to the body in multiple participants. This typically resulted in loss of contact between the pelvis brace and pelvis, and therefore in loss of transfer of support. This would explain the difference in generated and command torque in R2.0 (Figure 5). Note that the torque tracking in conditions with no shifting (e.g., R0.5, R0.8) was generally satisfactory (Figure 5). In addition, the shifting resulted in a considerable sagittal plane joint centre misalignment of the exoskeleton lumbar and L5S1 joints in R2.0 and LF relative to MINIMP, R0.8, and R0.5 (Table S1, Figure S4).

### 3.2. Effect of exoskeleton support with different L/H support ratios on spinal loading

**Figure 6.**
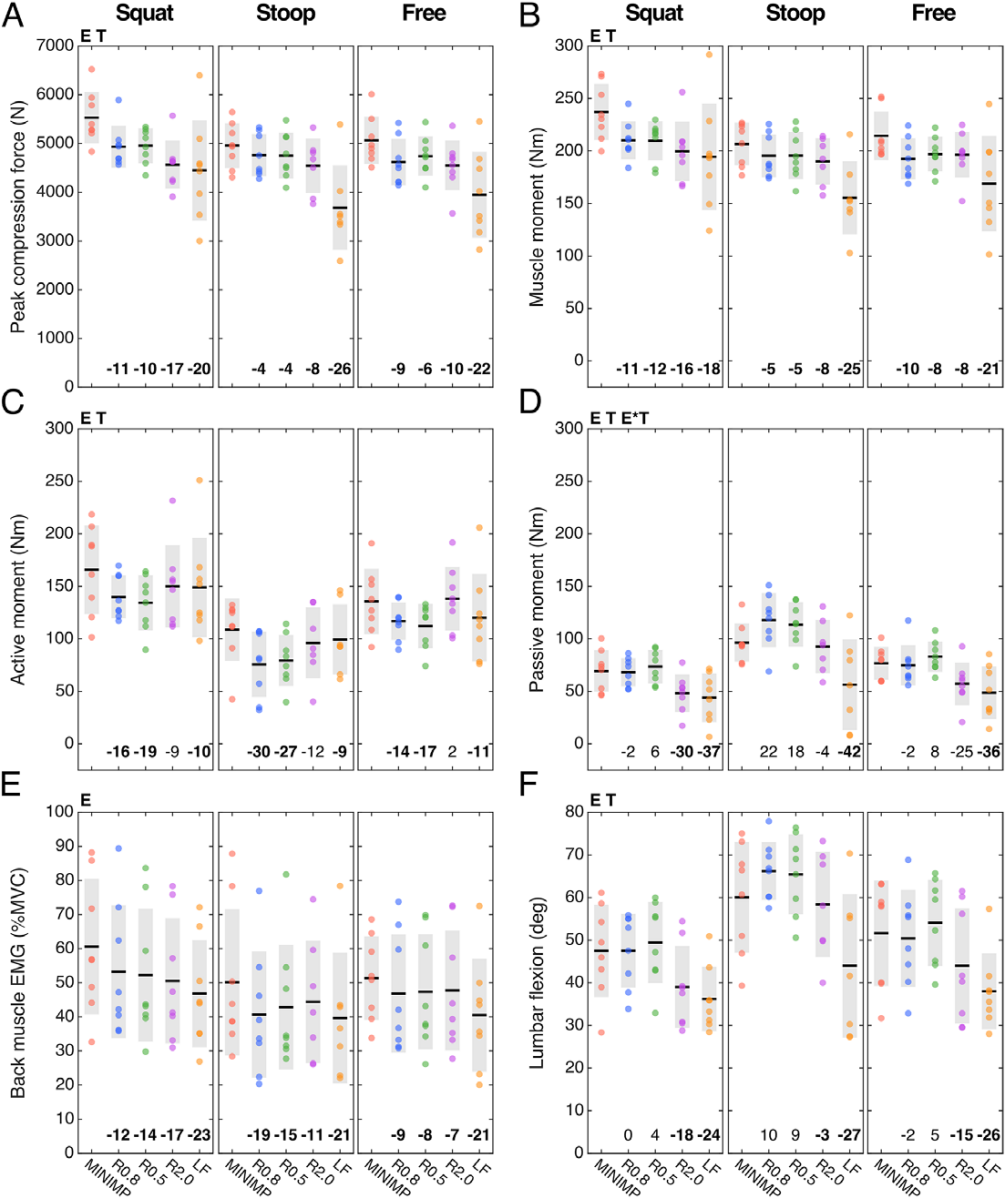
Peak compression force and muscle, active, and passive moment, back muscle EMG, and lumbar flexion at peak compression force (A-F, respectively) per technique and for the minimal impedance (MINIMP) and exoskeleton support conditions (see Table 1 for conditions). If applicable, significant main and interaction effects are indicated at the top of each subfigure (E: main exoskeleton effect; T: main technique effect; E*T: exoskeleton*technique interaction effect). The black line and grey rectangle depict the mean and standard deviation, respectively. The percentage difference in mean relative to MINIMP is presented at bottom of each graph, in bold when significant (Tables 2 & 3).

For peak Fcomp we found a main exoskeleton effect, but no interaction effect between exoskeleton and technique (Table 2). Compared to MINIMP, across lifting techniques significantly reduced peak Fcomp was found for all conditions (R0.8: 4–11%; R0.5: 4–10%; R2.0: 8–19%; LF: 20–26%; Table 3; Figure 6A). Among R0.8, R0.5, and R2.0 no significant differences were found in peak Fcomp, whereas Fcomp was significantly lower in LF compared to all other conditions (note however that that in LF lifting height was adjusted for three participants). Mmusc at peak Fcomp yielded similar statistical results and relative differences (Tables 2 and 3; Figure 6B).

**Table 2.** Effect of exoskeleton support with different L/H support ratios. *p*-Values of two-way ANOVA (with subject as random factor) with factors exoskeleton (MINIMP, R0.8, R0.5, R2.0, LF), technique (squat, stoop, and free) and their interaction. Significant *p*-values are presented in bold.

	Exoskeleton	Technique	Interaction
Peak compression force (Fcomp)	**<0.001**	**0.035**	0.147
Total muscle moment (Mmusc)	**<0.001**	**0.012**	0.281
Active moment (Mact)	**0.009**	**<0.001**	0.490
Passive moment (Mpas)	**<0.001**	**<0.001**	**0.006**
Lumbar flexion	**<0.001**	**<0.001**	0.068
Back muscle EMG	**<0.001**	0.181	0.661
Net L5S1 moment (Mnet)	0.125	0.285	0.060
Hip joint centre alignment error	**0.001**	0.580	0.055
Lumbar joint centre alignment error	**<0.001**	0.955	0.479

**Table 3.** Multiple comparisons for measures yielding a significant main effect for exoskeleton condition or, in case of Mpas, a significant interaction effect between exoskeleton and technique.

	R0.8	R0.5	R2.0	LF
Peak compression force (Fcomp)				
MINIMP	**<0.001**	**0.003**	**<0.001**	**<0.001**
R0.8		1	0.220	**<0.001**
R0.5			0.068	**<0.001**
R2.0				**<0.001**
Total muscle moment (Mmusc)				
MINIMP	**<0.001**	**0.001**	**<0.001**	**<0.001**
R0.8		1	1	**<0.001**
R0.5			1	**<0.001**
R2.0				**<0.001**
Active moment (Mact)				
MINIMP	**<0.001**	**<0.001**	0.325	**0.017**
R0.8		1	**0.005**	0.128
R0.5			**0.001**	**0.034**
R2.0				1
Passive moment (Mpas)				
*Squat (p < 0.001)*				
MINIMP	1	1	**0.022**	**0.004**
R0.8		1	**0.037**	**0.006**
R0.5			**0.004**	**<0.001**
R2.0				1
*Stoop (p < 0.001)*				
MINIMP	0.356	0.846	1	**0.006**
R0.8		1	0.246	**<0.001**
R0.5			0.582	**<0.001**
R2.0				**0.015**
*Free (p < 0.001)*				
MINIMP	1	1	0.100	**0.005**
R0.8		1	0.189	**0.010**
R0.5			**0.011**	**<0.001**
R2.0				1
Lumbar flexion				
MINIMP	1	0.178	**<0.001**	**<0.001**
R0.8		1	**<0.001**	**<0.001**
R0.5			**<0.001**	**<0.001**
R2.0				**<0.001**
Back muscle EMG				
MINIMP	**<0.001**	**<0.001**	**<0.001**	**<0.001**
R0.8		1	1	**0.035**
R0.5			1	**0.012**
R2.0				**0.027**

For Mact and lumbar flexion at peak Fcomp, we found a main exoskeleton and no interaction effect, whereas for Mpas an interaction effect of exoskeleton with technique was found (Table 2). With respect to the distribution of Mmusc between Mact and Mpas at peak Fcomp, R0.8 and R0.5 yielded a significantly reduced Mact (14–30% and 17–27%, respectively; Table 3, Figure 6C) with no difference in Mpas (Table 3, Figure 6D) and lumbar flexion (Table 3, Figure 6F) when compared to MINIMP. Although not significant, for these conditions a slight increase in lumbar flexion, and consequently Mpas, appeared to explain part of the decrease in Mact during stoop lifting. In R2.0, Mact was not different from MINIMP, partly due to a significantly reduced lumbar flexion (3–18%), requiring additional Mact to counteract Mnet. In LF, Mact, Mpas, and lumbar flexion were significantly reduced relative to MINIMP (9–11%, 36–42%, 24–27%, respectively). Mact, Mpas, and lumbar flexion were not different between R0.8 and R0.5, indicating a similar load distribution between active and passive tissues for these conditions. Lumbar flexion of both R2.0 and LF were significantly lower relative to all other conditions, with LF yielding the lowest lumbar flexion. In line, LF generally yielded a lower Mpas relative to other conditions.

Multiple comparisons for back EMG at peak Fcomp revealed a significantly reduced EMG amplitude for all exoskeleton conditions versus MINIMP, as well as a significantly reduced EMG amplitude for LF compared to all other conditions (Table 3, Figure 6E).

No exoskeleton or interaction effect was found on Mnet, suggesting that lifting technique at peak Fcomp was not significantly different between exoskeleton conditions (Table 2; Figure S5).

## 4. Discussion

In this study, we evaluated the effect of four exoskeleton support conditions with different L/H support ratios on spinal loading, lumbar kinematics, and back muscle EMG. We used a novel exoskeleton including two pairs of bilateral actuated joints, allowing separate lumbar and hip support, and subject-specific exoskeleton control, only supporting the moment actively generated by the back muscles to counteract the moment caused by the gravitational force acting on the upper body. Compared to MINIMP, peak Fcomp was on average significantly reduced in all exoskeleton support conditions. However, in R2.0 and LF lumbar flexion was clearly restricted, whereas in R0.8 and R0.5 the lumbar flexion angle was not significantly different from MINIMP. This indicates that when the L/H support ratio is in line with the L/H Mnet ratio during lifting (Figure S1), spinal loading can be reduced with exoskeleton support while allowing normal lifting behavior. In contrast, with a considerably higher L/H support ratio than the typical L/H Mnet ratio during lifting, for example, in case of an exoskeleton with only one bilateral pair of actuated hip joints and a rigid pelvis-trunk structure (i.e., LF), spinal loading can be reduced, but lumbar flexion is constrained, most likely hampering intended lifting behavior. This is typically experienced as a hinderance (Näf et al., 2018) and may therefore negatively affect user acceptance. In addition, in LF three participants were not able to reach the box with one or more techniques, highlighting the severe restriction of lumbar range of motion in this condition. For these trials, the required elevation of the box may have reduced the lumbar flexion and trunk inclination (i.e., Mnet) at the moment of box lifting. However, overall no exoskeleton effect on Mnet was found (Table 2, Figure S5), suggesting the effect of elevating the box in these trials on the average reduction in peak Fcomp and Mmusc in LF when compared to MINIMP was likely limited.

In comparing reductions in peak Fcomp and Mmusc at peak Fcomp across exoskeleton support conditions, one should consider between-condition differences in lumbar support, since this was not standardized in the current study. Obviously, for a given magnitude of hip support, a higher L/H support ratio results in a higher magnitude of lumbar support, and consequently in a greater reduction in spinal musculoskeletal loading. Although not measured, a considerably higher lumbar support would explain the significantly higher reduction in Fcomp and Mmusc in LF when compared to R0.8, R0.5, and R2.0. Across R0.8, R0.5, and R2.0, the average measured lumbar support at peak Fcomp was generally similar (across techniques: 19–35; 17–26; 18–21 Nm, respectively, Figure 3A), and no significant differences in peak Fcomp and Mmusc were found, while lifting behavior differed.

No clear differences in peak Fcomp, Mact-Mpas distribution, lumbar flexion, and back muscle EMG were found between R0.8 and R0.5. Since the magnitude of support in R0.8 and R0.5 was similar, differences were likely difficult to reveal with the current sample size. However, based on the general pattern of Mact, Mpas, and lumbar flexion in squat and free lifting between R0.8 and R0.5 (Figure 6), we would argue that an L/H ratio lower than 0.5 may result in increased lumbar flexion and Mpas when compared to MINIMP, risking damage to passive tissues (Solomonow et al., 1999).

When compared to MINIMP, R2.0 yielded similar lumbar flexion reductions during free lifting (15%) as a previous passive exoskeleton, which included a flexible beam aimed at improving range of motion (approx. 15%; Koopman, Näf, et al., 2020; Näf et al., 2018). For LF, the reduction in lumbar flexion at peak Fcomp (24–27%) was in line with previous research on an active exoskeleton with only one actuated hip DOF (33%; Koopman, Toxiri, et al., 2019). Koopman et al. (2019) reported an average reduction in peak Fcomp of 18%, less than in the present study (20–26%), probably due to differences in the magnitude of hip support at peak Fcomp (Koopman, Toxiri, et al., 2019: approx. 10-20 Nm; this study: 30–37 Nm, Figure 3A). In line with this study, one previous study suggested that in stoop lifting an exoskeleton including two pairs of bilateral actuators (at hip and low-lumbar levels) may restrict lumbar flexion less than a design including only one pair of bilateral actuated hip joints (Schwartz et al., 2023). However, this study did not report the L/H support ratio. Other studies, all on active exoskeletons including one bilateral pair of actuated hip joints, reported reductions in EMG amplitude, however did not report (lumbar) kinematics or use musculoskeletal modelling to estimate muscle or spinal loading (Chen et al., 2018; Heo et al., 2020; Huysamen et al., 2018; Hyun et al., 2020; Toxiri et al., 2018; Walter et al., 2023; Yamanaka et al., 2021). More thorough evaluation, using for example EMG-driven musculoskeletal modeling to account for the relationship between EMG and muscle force, is required for these previous active exoskeletons, to accurately assess the effect of exoskeleton support on musculoskeletal loading, as previously recommended (Kingma et al., 2022; Theurel & Desbrosses, 2019). Indeed, the results of the current study do suggest that the relationship between EMG and muscle force should be considered. For example, in R0.8 and R0.5, the reduction in back muscle EMG amplitude during stoop lifting was not proportional to the reduction in Mact or peak Fcomp, due to the considerable contribution of the Mpas. In addition, when compared to MINIMP, the back muscle EMG amplitude was significantly reduced in R2.0 whereas Mact was not significantly reduced.

In multiple participants, we observed considerable shifting of the pelvis brace during R2.0 and LF, which may have resulted in insufficient transfer of support (Figure 5) or, in some cases, support other than intended due to interference between the pelvis brace and trunk or pelvis IMU. Since shifting suggests the exoskeleton is unable to follow the intended motion and in MINIMP, R0.8, and R0.5 no shifting was observed, the shifting observed in R2.0 and LF suggests these conditions imposed an L/H support ratio not in line with the intended lifting behaviour. Shifting would also be problematic in exoskeletons relying on internal sensors (e.g., in estimating joint angles, a greater misalignment would result in a greater difference between exoskeleton and human joint angles). Furthermore, although Figure S4 suggests that exoskeleton-anatomical joint alignment error may in part be related to the magnitude of support at the respective joint, shifting of the pelvis brace in R2.0 and LF resulted in a considerable lumbar joint misalignment. In R0.8 and R0.5 no shifting was observed and lumbar joint misalignment was generally limited, suggesting with these L/H support ratios, the exoskeleton fit and transfer of support was better than in R2.0 and LF.

The exoskeleton control in the current study was based on a subject-specific model estimating the actively generated moment to counteract the moment caused by the gravitational force acting on the upper body, as suggested by Tabasi et al. (2020). This control reduces the support when the moment generated by passive forces increases (see Figure 4), to prevent counterproductive support during this phase; bending down would then require abdominal muscle activity, increasing spinal loading. The variance in support at peak Fcomp across participants during stoop lifting (Figure 3A) suggests the need for such a subject-specific exoskeleton control model. In one participant during squat and free lifting, the support approached zero, indicating that during these techniques Mpas was (almost) equal to Mnet_ub_ (Figure 3A). In previous active exoskeletons, the control has been based mainly on support proportional to trunk inclination (Hara and Sankai, 2010; Ko et al., 2018; Koopman, Toxiri, et al., 2019; Lazzaroni et al., 2020; Toxiri et al., 2018). Some studies have also considered the external load, due to handling the box, in exoskeleton control based on forearm (Koopman, Toxiri, et al., 2019) or low-back (Hara and Sankai, 2010) muscle EMG. In this study, the external load was not included in exoskeleton control, resulting in minimal requirements regarding calibration, sensors, and real-time computation. The torque-generating capacity of the current exoskeleton would allow including support related to the external load which could potentially further reduce peak Fcomp. This would, however, require more complex control, sensors and calibration (Moya-Esteban et al., 2022; Tabasi et al., 2020, 2022).

Wearing the exoskeleton without support (i.e., MINIMP) resulted in no difference in Mmusc, but a significant increase in lumbar flexion, and consequently redistribution of Mact and Mpas, when compared to NOEXO. This increase in lumbar flexion could be explained by a the dorsally located mass of the exoskeleton on the pelvis, inducing a backward pelvis tilt. We acknowledge that the current exoskeleton mass is considerably higher than that of other exoskeleton designs. However, the current version of this exoskeleton should be considered a proof of concept, where we favored torque-generating capacity and a rigid structure over a light-weight design.

The performance of the fitted EMG-driven musculoskeletal model estimated using the RMSE and *R* ^2^ between Msubject and Mmusc (18.8–26.3 Nm and 0.75–0.93, respectively) was similar to previous studies (Koopman, Toxiri, et al., 2019; Tabasi et al., 2022). Average peak Fcomp was also similar to previous research on lifting with a 15 kg load (Koopman, Kingma, et al., 2020; Koopman, Toxiri, et al., 2019).

In addition to before-mentioned limitations, others should be mentioned. Between-condition differences in familiarity with the provided exoskeleton support with L/H support ratio should be considered. Exoskeleton familiarization included only trials involving MINIMP and R0.8 and in all participants the MINIMP, R0.8, and R0.5 trials were recorded before R2.0 and LF, to limit the risk of affecting the instrumentation due to exoskeleton pelvis brace shifting. This may have resulted in less familiarity for R2.0 and LF when compared to the other conditions. On the other hand, the required familiarization may also be different between conditions. For this study, familiarization was limited and between-condition differences in familiarity are difficult to estimate. The present results only indicate acute effects of exoskeleton support with different L/H support ratios.

The user’s dynamics were not included in the exoskeleton control. Including trunk angular acceleration in exoskeleton control has been suggested. However its benefit relative to control proportional to trunk inclination could not be found, possibly due to torque-generating limitations (Lazzaroni et al., 2020). In addition, it should be noted that, in the current calibration of the exoskeleton control model, the assumption that Mnet_ub_ during full flexion was completely generated by Mpas may only apply to individuals demonstrating flexion relaxation during full flexion, but not to individuals demonstrating no flexion relaxation (Laird et al., 2018).

Moving forward, the L/H support ratio could be adjusted in real-time based on an estimation of the L/H Mnet ratio to better support intended motion across users and tasks. In addition, the current exoskeleton design would allow interventions involving changes in L/H flexion ratio to, for example, impose subtle changes in lifting behaviour or delay back muscle fatigue during prolonged bending (Brouwer et al., 2024). The findings in this study may also guide improving passive and semi-active exoskeleton design (e.g., through addition of a lumbar joint) or support to better accommodate the user’s intended motion. Lastly, the weight of the exoskeleton could be reduced to limit the load on participants.

## 5. Conclusions

We developed a novel active exoskeleton including two bilateral pairs of actuated joints, allowing separate lumbar and hip support, and exoskeleton control providing support as a percentage of the low-back moment actively generated by the back muscles to counteract the moment caused by the gravitational force acting on the upper body. We evaluated the effect of exoskeleton support with different lumbar-to-hip (L/H) support ratios on spinal loading, lumbar kinematics, and back muscle EMG during lifting loads with three techniques. The support provided at peak spinal loading indicated considerable between-participant differences in distribution between actively and passively generated moments, highlighting the need for subject-specific exoskeleton control. The results also indicate that an L/H support ratio in line with the typical user’s L/H net moment ratio reduces spinal loading, while allowing intended lifting behaviour, possibly increasing acceptance. In contrast, considerably higher L/H support ratios, as in exoskeletons with only one bilateral pair of actuated hip joints combined with a rigid structure between the pelvis and trunk, reduce spinal loading while changing lifting behaviour (i.e., considerably restricting lumbar flexion). This restriction of intended lifting behaviour is typically experienced as hindrance and may prevent task completion, possibly hampering acceptance. Finally, the results highlight the importance of taking into account factors associated with the EMG–muscle force relationship, such as lumbar flexion, when evaluating exoskeletons.

## Supporting information

Brouwer et al. supplementary material Brouwer et al. supplementary material

## Data Availability

The data that support the findings of this study are available from the corresponding author upon reasonable request.

## Supplementary material

The supplementary material for this article can be found at http://doi.org/10.1017/wtc.2024.7.
