# Supplementary material for: The effect of active exoskeleton support with different lumbar-to-hip support ratios on spinal musculoskeletal loading and lumbar kinematics during lifting
Source: Wearable Technol. 2024 Dec 23;5:e25. doi: 10.1017/wtc.2024.7 (PMC11729479; doi:10.1017/wtc.2024.7)
Supplement: Brouwer et al. supplementary material [file S2631717624000070sup001.pdf]

Supplementary Data

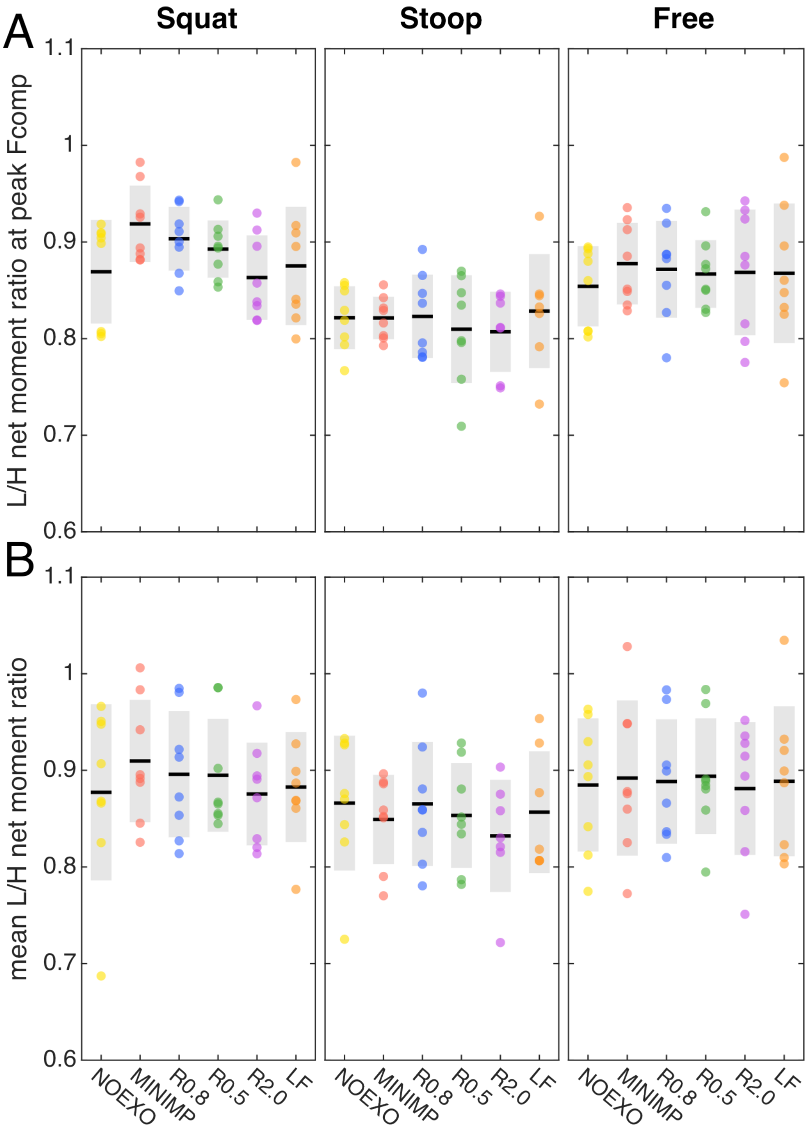

Figure S1. *(A) lumbar-to-hip (L/H) net moment ratio at peak compression force (Fcomp); (B) mean L/H net moment ratio across trial. Note that the lumbar net moment is the L5S1 net moment. The black line and grey rectangle depict the mean and standard deviation, respectively.*

Table S1. *Step 1: effect of wearing exoskeleton without support. P-values of repeated measures ANOVA with factors Exoskeleton (NOEXO MINIMP), Technique (squat, stoop, and free) and their interaction. Significant p-values are presented in bold.*

	Exoskeleton	Technique	Interaction
Peak compression force (Fcomp)	0.681	0.052	0.236
Total muscle moment (Mmusc)	0.637	**0.024**	0.478
Active moment (Mact)	**0.005**	**0.003**	0.217
Passive moment (Mpas)	**0.001**	**0.003**	**0.009**
Lumbar flexion	**<0.001**	**0.003**	**0.010**
Back muscle EMG	0.299	0.165	0.545
Net L5S1 moment (Mnet)	**0.020**	0.073	0.062

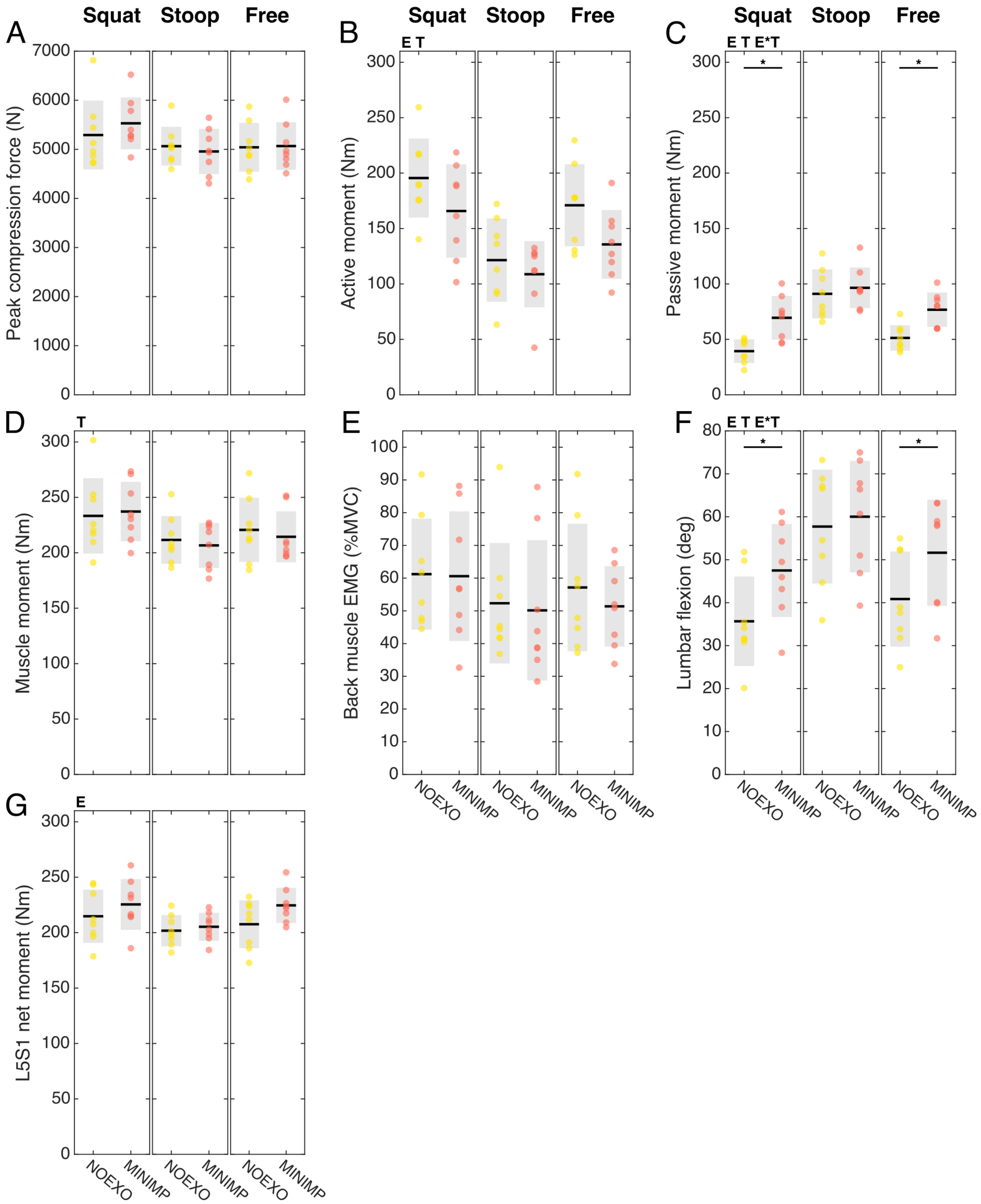

Figure S2. *Peak compression force and active, passive, muscle moment, back muscle EMG, lumbar flexion, and Net L5S1 moment at peak compression force (A-G, respectively) per technique and for the without-exoskeleton (NOEXO) and minimal impedance (MINIMP) conditions. If applicable, significant main and interaction effects are indicated at the top of each subfigure (E: main exoskeleton effect; T: main technique effect; E*T: exoskeleton*technique interaction effect). In case of E*T, post-hoc significant differences per technique are depicted using an asterisk. The black line and grey rectangle depict the mean and standard deviation, respectively.*

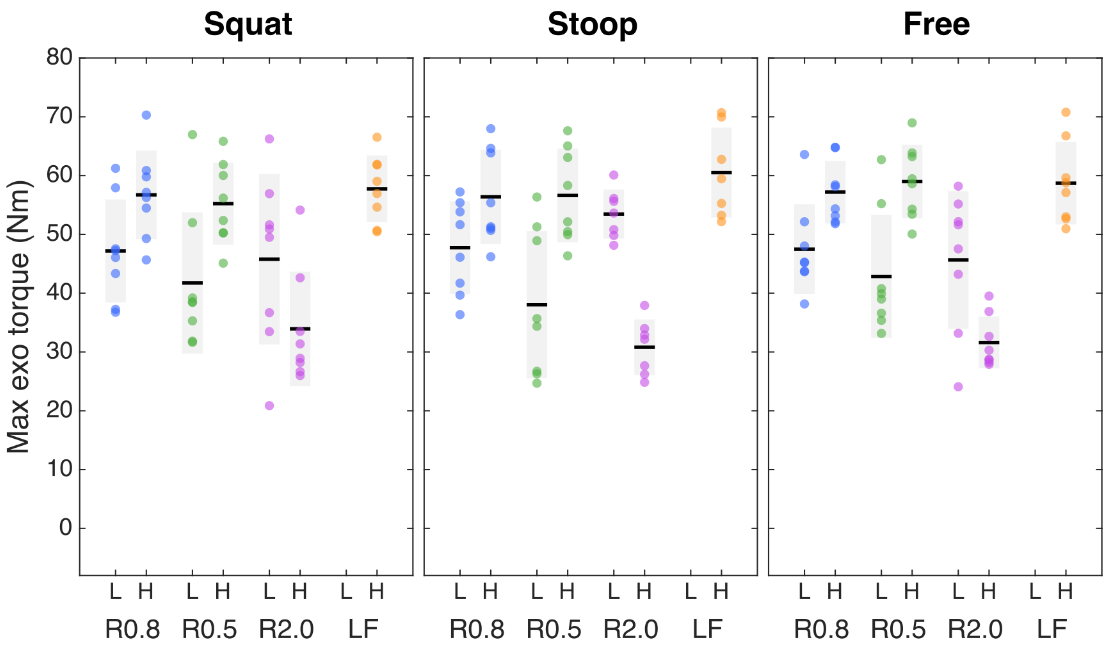

Figure S3. *Maximum generated lumbar (L) and hip (H) support across trial, for the conditions involving exoskeleton support, per lifting techniques. Note that for LF no lumbar torque was included since the lumbar exoskeleton joint was mechanically fixed. The black line and grey rectangle depict the mean and standard deviation, respectively.*

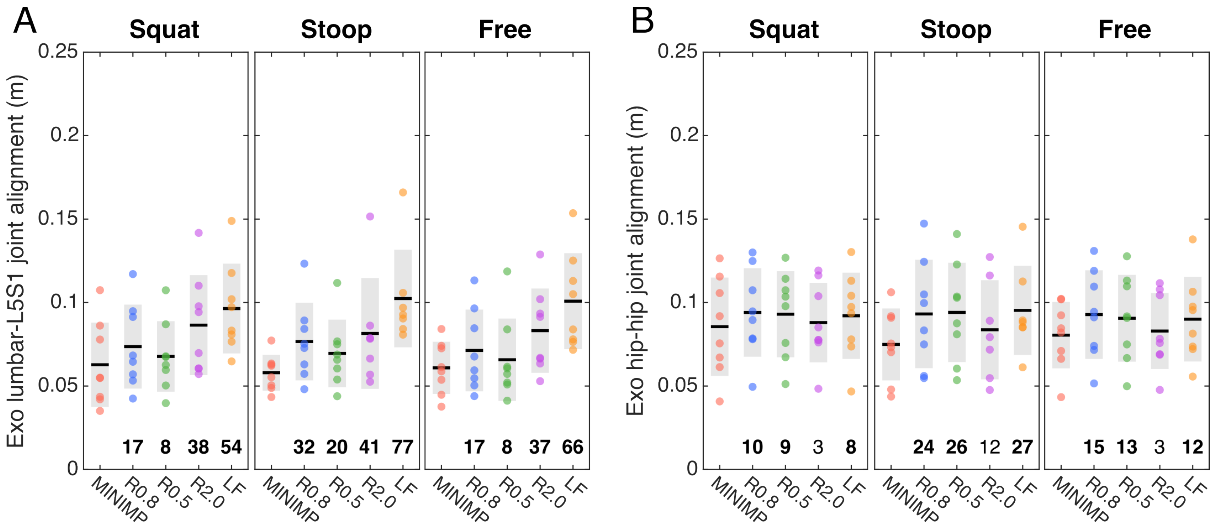

Figure S4. *Sagittal plane alignment error of (A) exoskeleton (Exo) left/right lumbar joint with respect to the L5S1 joint; and (B) Exo left/right hip joints with respect to left/right hip joints (root-mean-square of sagittal plane vector norm), per technique and for the minimal impedance (MINIMP) and exoskeleton support conditions (see Table 1 for conditions). The black line and grey rectangle depict the mean and standard deviation, respectively. The percentage difference in mean relative to MINIMP is presented at bottom of each graph, in bold when significant in case of significant main exoskeleton effect (Table S2).*

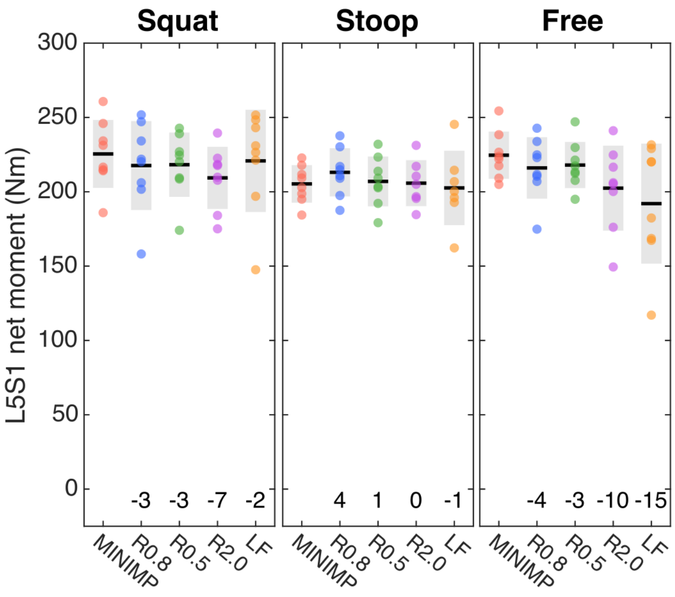

***Figure S5**. Net L5S1 moment (Mnet) at peak compression force per technique and for the minimal impedance (MINIMP) and exoskeleton support conditions. The black line and grey rectangle depict the mean and standard deviation, respectively.*

Table S2. *Step 2 multiple comparisons for sagittal plane exoskeleton-anatomical joint alignment error yielding a significant exoskeleton effect.*

		R0.8	R0.5	R2.0	LF
Hip joint alignment error					
	MINIMP	**<0.001**	**<0.001**	0.422	**<0.001**
	R0.8		1	**<0.001**	1
	R0.5			**<0.001**	1
	R2.0				**<0.001**
Lumbar joint alignment error					
	MINIMP	**<0.001**	**0.025**	**<0.001**	**<0.001**
	R0.8		0.084	**0.001**	**<0.001**
	R0.5			**<0.001**	**<0.001**
	R2.0				**<0.001**
